# Outcomes of computer-assisted peri-acetabular osteotomy compared with conventional osteotomy in hip dysplasia

**DOI:** 10.1007/s00264-020-04578-x

**Published:** 2020-04-28

**Authors:** Hiroshi Imai, Tomomi Kamada, Joji Miyawaki, Akira Maruishi, Naohiko Mashima, Hiromasa Miura

**Affiliations:** Department of Bone and Joint Surgery, Ehime University Graduate School of Medicine, Shitsukawa, Toon, Ehime 791-0295 Japan

**Keywords:** Developmental dysplasia of the hip, Computer-assisted periacetabular osteotomy, Clinical and radiographic outcomes, Adequate anterior and lateral acetabular coverage

## Abstract

**Aim of the study:**

To compare the outcomes after computer-assisted peri-acetabular osteotomy (PAO) and conventional PAO performed for hip dysplasia (DDH).

**Methods:**

Ninety-one patients (98 hips) were enrolled in this study. In each case, DDH was treated with either conventional PAO, in which the angle and direction of the osteotomy was determined by intra-operative X-ray examination, or with computer-assisted PAO, which used the 3D navigation system. Forty hips underwent conventional PAO and 58 hips underwent computer-assisted PAO.

**Results:**

Japanese Orthopaedic Association hip scores improved significantly from 70.0 points pre-operatively to 90.7 points post-operatively in patients with conventional PAO, and from 74.5 points pre-operatively to 94.2 points post-operatively in patients with computer-assisted PAO. In all patients with computer-assisted PAO, the post-operative AHI and VCA angle were within the radiographic target zone. Some patients with conventional PAO had post-operative AHI and VCA angle outside of the target zone. We performed total hip arthroplasty (THA) on five of the 98 PAO hips (5.1%) after an average follow-up period of 5.4 years. None of 58 hips (0%) with computer-assisted PAO was revised.

**Discussion:**

Computer-assisted PAO enabled intra-operative confirmation of osteotomy sites, and the position of the osteotomized bone fragment could be confirmed in real time. Adequate anterior and lateral coverage of the femoral head in patients with computer-assisted PAO resulted in no need for early conversion to THA, in contrast to conventional PAO.

**Conclusion:**

Computer-assisted PAO not only improved accuracy and safety but also achieved sufficient anterior and lateral displacement to prevent the progression of DDH.

## Introduction

Developmental dysplasia of the hip (DDH) is a frequent cause of secondary osteoarthritis (OA). It is also the leading cause of end-stage OA of the hip in patients younger than 50 years. Peri-acetabular osteotomy (PAO), which includes rotational acetabular osteotomy (RAO) [1], eccentric rotational acetabular osteotomy (ERAO) [2], Bernese peri-acetabular osteotomy (PAO), and curved peri-acetabular osteotomy (CPO) [3, 4], aims to correct the deficient acetabular coverage in hips with DDH so as to prevent secondary OA.

It is difficult, however, to obtain adequate acetabular coverage of the femoral head after PAO. Because insufficient anterior or lateral coverage of the femoral head during PAO can cause progressive osteoarthritic changes, excessive anterior displacement added to lateral displacement can impair activities of daily living by limiting the range of motion (ROM) of the hip joint, and pincer femoroacetabular impingement (FAI) syndrome can occur [5, 6].

To improve the accuracy and safety of complex orthopedic surgeries, computer-assisted techniques have recently been introduced [7]. We began performing PAO for DDH using computer-assisted techniques beginning in June 2013.

The purposes of this study were to compare the intermediate-term clinical and radiographic outcomes of computer-assisted and conventional PAO performed for DDH in young adults.

## Materials and methods

From December 2007 to February 2019, we performed PAO in patients with symptomatic DDH and pain. Of 127 consecutive patients (152 hips) who underwent PAO and could be followed up for three years or more, 91 patients (98 hips) were recruited for this study. The following patients were excluded: 33 (51 hips) who were followed up for less than three years, two (two hips) who did not attend all follow-up appointments, and one (one hip) who was lost to follow-up. The mean age of the subjects at the time of the operation was 39.1 years (15 to 56). There were 11 male patients (15 hips) and 80 female patients (83 hips). The mean duration of follow-up after the operation was 5.4 years (3 to 11). The mean BMI was 22.6 kg/m^2^ (16.7 to 37.8).

DDH was classified using the Tönnis et al. classification. Based on this classification, 27 hips in this study had grade 0 DDH, 60 had grade 1, 11 had grade 2, and none had grade 3 [8]. Seven patients (seven hips) had undergone previous surgery during infancy to reduce congenital dislocation of the hip.

All operations were performed by one senior surgeon. PAO was performed according to the technique described by Hasegawa [2]. The patient was positioned in the lateral position. The greater trochanter was detached with an oscillating saw and was reflected proximally. A curved osteotomy chisel was introduced proximately 20 mm superior to the joint space, and an eccentric osteotomy was made. All acetabular osteotomies were performed using a curved chisel with a 40- or 45-mm radius.

In conventional PAO from December 2007 to May 2013, the angle and direction of the osteotomy were determined by intra-operative X-ray. Rotation of the acetabular fragment allowed for simultaneous medial and distal displacement of the femoral head. The osteotomized acetabular fragment was moved laterally and about 1 cm anteriorly to obtain superior lateral and anterior coverage of the femoral head. In terms of radiographic findings, a lateral center edge (LCE) angle of > 25° is necessary to obtain adequate superolateral coverage of the femoral head [9].

**Fig. 1:**
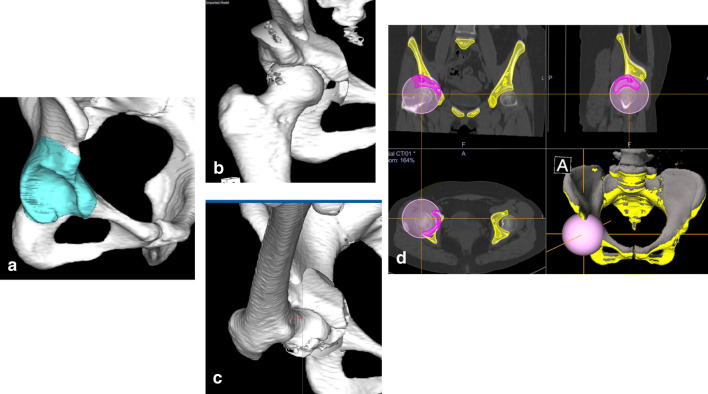
**a**–**c** Three-dimensional preoperative planning with the Zed HIP® planning software is converted to the standard template library (STL) format. **d** The STL format is transferred to the OrthoMap® 3D navigation software and the CT-based hip navigation system (Stryker Orthopaedics, Mahwah, NJ, USA)

In computer-assisted PAO beginning in June 2013, we performed three-dimensional reconstruction using the Zed HIP® planning software (LEXI, Tokyo, Japan) and moved the osteotomized acetabular fragments laterally and anteriorly to obtain at least 75% coverage of the femoral head for DDH (Fig. 1a, b). This coverage was calculated using the functional pelvic plane [10]. The angle and direction of the osteotomized acetabular fragment, as determined with the Zed HIP® planning software, were implemented intra-operatively using the OrthoMap® 3D Navigation software (Stryker, Freiburg, Germany) and the CT-based hip navigation system (Stryker Orthopaedics, Mahwah, NJ, USA). Adequate anterior and lateral displacement is important to avoid FAI at 110° or less of flexion, as shown by the range of motion simulation using this software (Fig. 1c, d). Therefore, the approximate post-operative radiographic target zone after PAO for DDH was 60° or less, in addition to 20° or more of the vertical axis centre of femoral head–anterior extremity of acetabular roof angle (VCA) in the false profile view [6, 11].

Intra-operatively, three pins were inserted into the iliac crest through a separate small incision and the pelvic tracker was placed. The screen of the CT-based hip navigation system was positioned 1 m away from the operation table at the cranial end of the patient; the correlation between the pre-operative image data and the patient’s anatomy was established by surface matching of 30 or more points. Registration was confirmed by capturing the bone surfaces with the tracked probe. After registration of the pelvis was completed, the osteotomy line in the pelvis could be checked on the screen of the navigation system, and the acetabulum was osteotomized according to the pre-operative plan. The osteotomized acetabular fragment was moved laterally and anteriorly as displayed intra-operatively on the preoperative three-dimensional plan projected onto the screen of the navigation system. After the fragment was displaced, four to five hydroxyapatite fixation screws were used for fragment fixation. The demographic data of the two patient groups are summarized in Table 1.

**Table 1:** Patient demographic data

	Conventional PAO (40 hips)	Computer-assisted PAO (58 hips)	*p* values
Age (years) ^c^	41.1 ± 1.5 (22–56)	37.7 ± 1.4 (15–52)	N.S.^a^
BMI (kg/m^2^) ^c^	23.0 ± 0.6 (16.7–32.2)	23.5 ± 0.5 (17.7–37.8)	N.S.^a^
Male/female	7/33	8/50	N.S.^b^
Follow-up period (years) ^c^	7.3 ± 0.2 (6–11)	4.1 ± 0.1 (3–6)	< 0.05^a^
Tönnis G0/1/2/3	10/23/7/0	17/37/4/0	N.S.^b^
Previous surgery	2/38	3/55	N.S.^b^

^a^Unpaired *t* test

^b^Chi-square test

^c^Values are expressed as the mean ± standard error, with range in parentheses

The Japanese Orthopaedic Association (JOA) scoring system was used to evaluate hip joint function [12] and investigate the incidence of post-operative complications. The JOA system consists of a 100-point scale comprising the following subcategories: pain (0–40 points), ability to walk (0–20 points), range of motion (0–20 points), and ability to complete tasks of daily living (0–20 points). Higher scores indicate better function. Scores at the final follow-up were compared with those obtained pre-operatively.

Radiographic examination was performed pre-operatively and at the final post-operative follow-up to calculate the LCE angle [9], sharp angle [13], acetabular-head index (AHI), and VCA angle in the false profile view [11].

Survival was evaluated by the Kaplan–Meier analysis [14] with failure as the endpoint, defined as conversion to THA. Log-rank tests were performed to determine whether conventional and computer-assisted PAOs were related to the risk of conversion to THA.

Three-dimensional CT scans were performed using a Philips Brilliance 64 scanner (Marconi Medical System, Best, Netherlands). All raw CT scan data were entered in the Digital Imaging and Communications in Medicine (DICOM) format into the Zed HIP® planning software and then converted to the standard template library (STL) format. The STL-formatted data were transferred to the OrthoMap® 3D Navigation software and the CT-based hip navigation system (Stryker Orthopaedics).

Clinical assessment and radiographic measurements were completed twice by two orthopedic surgeons, each with more than 15 years of experience in assessing hip function. Both surgeons were blinded to the radiographic results at the time of the evaluation. The time between measurements was at least two weeks. Intra- and inter-observer variances were calculated.

### Statistical analysis

The normality of continuous data was assessed with Levene’s test. Since the data were normally distributed, unpaired Student’s *t* test was used. Intra-observer variances in the JOA hip score were determined by comparing separate radiographic assessments of the same patient by the same observer with at least a 2-week interval between assessments. Intra-observer and inter-observer variances in the JOA hip score were determined by comparing radiographic measurements and are expressed using interclass correlation coefficients (ICC), with ICC < 0.20 indicating slight agreement, 0.21–0.40 fair agreement, 0.41–0.60 moderate agreement, 0.61–0.80 substantial agreement, and > 0.80 almost perfect agreement [15]. The survival rate was evaluated by the Kaplan–Meier analysis [14] as described above. The log-rank test was performed to determine whether conventional and computer-assisted PAOs were related to the risk of conversion to THA, with THA defined as described above for the Kaplan–Meier analysis.

SPSS for Windows version 20 (IBM Corp., Armonk, NY, USA) was used for all statistical analyses. A *p* value of < 0.05 was used to indicate statistical significance.

#### Ethics

This study was approved by our institution’s Ethics Committee and was conducted in accordance with the World Medical Association Declaration of Helsinki Standard of 1964, as revised in 1983 and 2000. All patients were informed about the study in detail before providing written informed consent for enrollment, including consent for post-operative computed tomography (CT) imaging.

## Results

The JOA hip scores improved significantly from 70.0 points pre-operatively (35 to 90) to 90.7 points post-operatively (50 to 100) in patients with conventional PAO, and from 74.5 points pre-operatively (55 to 93) to 94.2 points post-operatively (81 to 100) in patients with computer-assisted PAO.

Two intra-observer ICCs were calculated; both were 0.98. The inter-observer ICC was 0.86. These values indicate almost perfect agreement in JOA hip score measurements.

None of the patients developed post-operative infections, paralysis, deep vein thrombosis, or nonunion.

**Fig. 2:**
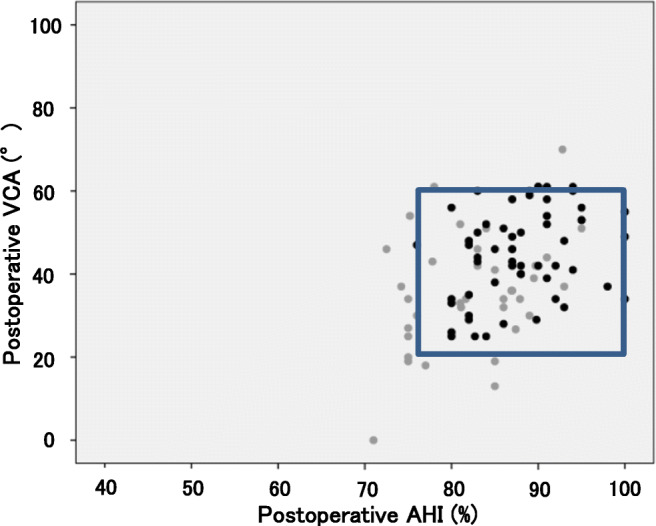
Scatter diagram of the postoperative AHI and VCA angle in patients with PAO for DDH. Data for computer-assisted PAO are shown using black markers. Data for conventional PAO are shown using gray markers

Radiographically, the post-operative AHI and VCA angle demonstrated significant differences between conventional and computer-assisted PAO (*p* < 0.05 and *p* < 0.05, respectively) (Table 2). In Fig. 2, black markers indicate patients who underwent computer-assisted PAO and whose postoperative AHI and VCA angle were within the radiographic target zone, while gray markers indicate patients who underwent conventional PAO and whose postoperative AHI and VCA angle were outside of the target zone.

**Table 2:** Comparison of intermediate-term clinical and radiographic outcomes after conventional and computer-assisted PAO for DDH

	Conventional PAO (40 hips)	Computer-assisted PAO (58 hips)	*p* values
JOA hip score (points) ^b^	90.7 ± 1.6 (50–100)	94.2 ± 0.6 (81–100)	N.S.^a^
LCE angle (°) ^b^	34.5 ± 1.1 (23–49)	37.4 ± 0.9 (25–50)	N.S.^a^
Sharp angle (°) ^b^	36.8 ± 0.6 (27–44)	36.0 ± 0.5 (26–43)	N.S.^a^
AHI (%) ^b^	82.7 ± 1.0 (71–95)	88.1 ± 0.7 (76–100)	< 0.05^a^
VCA angle (°) ^b^	36.5 ± 2.1 (0–70)	44.9 ± 1.5 (25–61)	< 0.05^a^

^a^Unpaired *t* test

^b^Values are expressed as the mean ± standard error, with range in parentheses

Two intra-observer ICCs for the radiographic measurements were calculated; both were 0.9 or more. The inter-observer ICCs were also 0.8 or more. These values indicate almost perfect agreement.

**Fig. 3:**
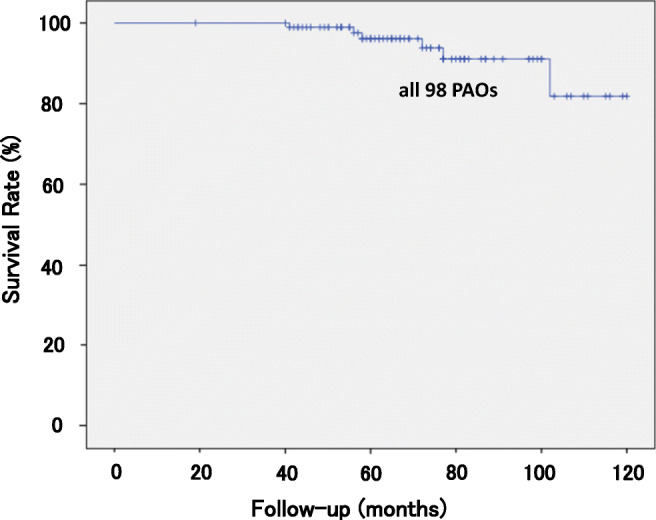
Kaplan–Meier survival analysis with conversion to THA as the endpoints

**Fig. 4:**
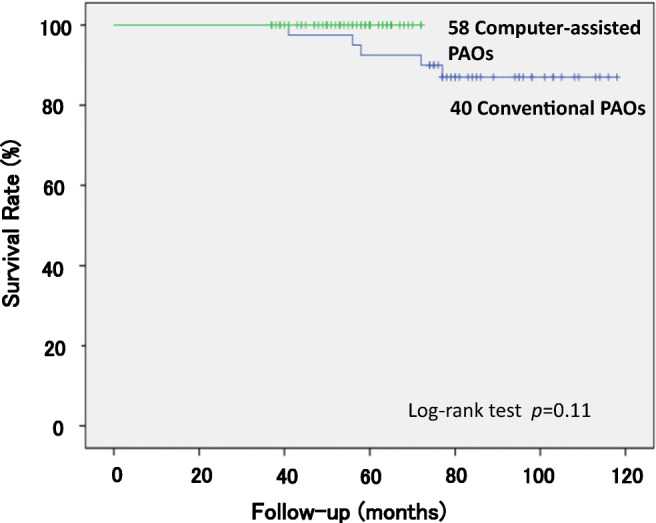
Kaplan–Meier survival analysis with conversion to THA as the endpoint, comparing conventional and computer-assisted PAOs.

A survival analysis was conducted for all 98 hips that underwent PAO. We performed THA on five hips (5.1%) after an average follow-up period of 5.4 years (3–11 years). The survivorship curves, with conversion to THA as the endpoint, are depicted in Fig. 3. The 11-year survival rate was 84.0%. None of the 58 hips that underwent computer-assisted PAO was revised (0%), compared with five of 40 hips that underwent conventional PAO (12.5%). Log-rank tests were performed to determine whether computer-assisted and conventional PAOs were related to the risk of conversion to THA, with THA defined as described above for the Kaplan–Meier analysis (*p* = 0.11) (Fig. 4). The two types of PAO did not differ in terms of their association with the risk of conversion to THA. However, no patients with computer-assisted PAO underwent early conversion to THA. We also performed log-rank tests to determine whether age at the PAO (older than 41 years vs younger than 40 years) was related to the risk of conversion to THA. No significant difference was observed between the patients older than 41 years (*n* = 47) and those younger than 40 years (*n* = 51) in terms of the follow-up period. Analysis with the log-rank tests revealed no significant difference between the survival rate of two groups (*p* = 0.16). And five hips with conversion to THA had not undergone previous surgery during infancy to reduce congenital dislocation of the hip.

## Discussion

DDH is one of the complex deformities in pediatric orthopedics and a frequent cause of secondary OA. Li Y et al. reported that residual acetabular dysplasia developed in 43.6% of the patients aged 24 to 36 months with DDH treated by closed reduction and spica cast immobilization in human position, secondary pelvic surgery without opening the joint usually provided good outcomes in most patients [16]. In terms of relationship between femoral anteversion angle (AA) and redislocation after closed reduction, Hong et al. reported that AA was different between affected and unaffected side of patients with unilateral DDH; however, the difference had very limited or no clinical significance because redislocation/sub-luxation was not influenced by AA values [17].

In this study, seven patients (seven hips) had undergone open reduction without pelvic or proximal femoral derotational osteotomy during infancy to reduce congenital dislocation of the hip. No cases of redislocation were observed after open reduction.

The deficient acetabular coverage in hips with DDH is the leading cause of end-stage OA of the hip. PAO aims to correct the deficient acetabular coverage in hips with DDH so as to prevent secondary OA in patients younger than 50 years. However, PAO is a complex surgical procedure with a substantial learning curve. Insufficient anterior or lateral coverage of the femoral head during the operation can cause progressive osteoarthritic changes, and the coverage of the femoral head by excessive anterior displacement may increase the incidence of post-operative FAI [5, 6, 18]. It is difficult to obtain adequate acetabular coverage of the femoral head after PAO. In a virtual osteotomy study using CT images of the hips with acetabular dysplasia, Dong Hun Suh et al. [19] suggested that when covering the femoral head, anterior displacement of the bone fragment is sometimes necessary in addition to lateral displacement. Siebenrock et al. [20] reported that pincer FAI occurred in 29% of the cases they examined after PAO. Since the introduction of the FAI concept, more emphasis has been placed on avoiding anterior and lateral overcorrection or retroversion, both of which may be associated with an unfavorable outcome.

To improve the accuracy and safety of these complex orthopaedic operations, computer-assisted techniques have recently been introduced [7]. They can enable three-dimensional pre-operative planning, intra-operative confirmation of osteotomy sites, and safe performance of osteotomy even under poor visual conditions. As a result, none of the patients developed post-operative infections, paralysis, deep vein thrombosis, or nonunion due to the large gap at the pubic osteotomy site 1 year after PAO [21–23]. And the favourable position of the osteotomized bone fragment [24] can be confirmed in real time with a tracking probe after the completion of osteotomy.

**Fig. 5:**
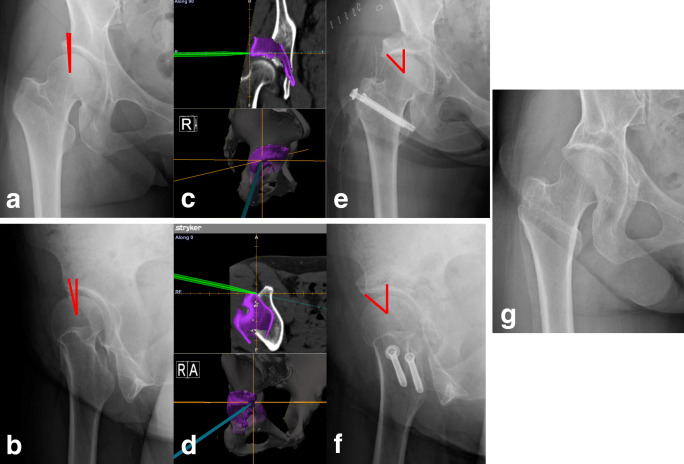
**a**, **b** The conventional AP pelvic radiograph and the false profile view of a 49-year-old woman with a dysplastic right hip with a pre-operative CE angle of 1° and a VCA angle of 12°. **c**, **d** Examination of the surface of the rotated bone fragment with a tracking probe after the completion of osteotomy, to determine whether the osteotomized acetabular fragment provided sufficiently lateral coverage and adequate anterior coverage. **e**, **f** The postoperative CE angle was 38° and the VCA angle was 42°. **g** At 5-year follow-up, no osteoarthritic changes were seen

We performed PAO in young adults with DDH using computer-assisted techniques beginning in June 2013 and compared the intermediate-term clinical and radiographic outcomes after conventional PAO with those after computer-assisted PAO. Computer-assisted PAO can be safely performed by checking the position of the tip of the curved chisel on the navigation screen, and using a tracking probe to confirm the position of the osteotomized bone fragment in real time after osteotomy completion (Fig. 5a–g). Our results showed that all patients with computer-assisted PAO demonstrated a postoperative AHI and VCA angle within the radiographic target zone. Adequate anterior and lateral coverage of the femoral head in patients with computer-assisted PAO resulted in no need for early conversion to THA, in contrast to conventional PAO.

Computer-assisted PAO not only results in improved accuracy and safety but also achieves adequate anterior and lateral displacement and thus prevents the progression of disease.

## Limitations

This study has several limitations. Firstly, it was retrospective in nature and represented a single surgeon’s experience in a high-volume centre. Secondly, 36 patients (54 hips) were excluded due to concurrent conditions, as detailed above. Thirdly, some patients who underwent computer-assisted PAO had a short follow-up period. Fourthly, we did not evaluate morphological variation of the anterior inferior iliac spine–affected bony ROM in simulated flexion after virtual PAO [25]. Finally, our conclusions were not fully definitive due to the small number of cases (*n* = 98) in this report.

## Conclusion

This study demonstrated positive intermediate-term clinical and radiographic outcomes after computer-assisted PAO for DDH. For the treatment of DDH, it is important to cover the femoral head by adequate anterior displacement to avoid FAI at flexion of 110° or less. Surgeons should also attain sufficient anterior and lateral displacement to prevent disease progression. Computer-assisted PAO can achieve these goals, and it is also both accurate and safe.

## References

[1] Ninomiya S, Tagawa H (1984). Rotational acetabular osteotomy for the dysplastic hip. J Bone Joint Surg Am.

[2] Hasegawa Y, Iwase T, Kitamura S, Yamauchi K, Sakano S, Iwata H (2002). Eccentric rotational acetabular osteotomy for acetabular dysplasia: follow-up of one hundred and thirty-two hips for five to ten years. J Bone Joint Surg Am.

[3] Ganz R, Klaue K, Vinh TS, Mast JW (1988). A new periacetabular osteotomy for the treatment of hip dysplasia: technique and preliminary results. Clin Orthop Relat Res.

[4] Naito M, Shiramizu K, Akiyoshi Y, Ezoe M, Nakamura Y (2005). Curved periacetabular osteotomy for treatment of dysplastic hip. Clin Orthop Relat Res.

[5] Ganz R, Parvizi J, Beck M, Leunig M, Nötzli H, Siebenrock KA (2003). Femoroacetabular impingement. Clin Orthop Relat Res.

[6] Imai H, Kamada T, Takeba J, Shiraishi Y, Mashima N, Miura H (2014). Anterior coverage after rotational acetabular osteotomy for the treatment of developmental dysplasia of the hip. J Orthop Sci.

[7] Amiot LP, Poulin F (2004). Computer tomography-based navigation for hip, knee, and spine surgery. Clin Orthop Relat Res.

[8] Tönnis D, Heinecke A (1999). Acetabular and femoral anteversion: relationship with osteoarthritis of the hip. J Bone Joint Surg Am.

[9] Wiberg G (1953). Shelf operation in congenital dysplasia of the acetabulum and in subluxation and dislocation of the hip. J Bone Joint Surg Am.

[10] Sugano N, Nishii T, Miki H, Yoshikawa H, Sato Y, Tamura S (2007). Mid-term results of cementless total hip replacement using a ceramic-on ceramic bearing with and without computer navigation. J Bone Joint Surg.

[11] Lequesne Par M, De Séze S: False profile of the pelvis (1961) A new radiographic incidence for the study of the hip-its use in dysplasias and different coxapathies (in French). Rev. Rhum Mal Osteoartic 28:643–652 14464207

[12] Mibe J, Imakiire A, Watanabe T, Fujie T (2005). Results of total hip arthroplasty with bone graft and support ring for protrusion acetabuli in rheumatoid arthritis. J Orthop Sci.

[13] Sharp IK (1961). Acetabular dysplasia: the acetabular angle. J Bone Joint Surg (Br).

[14] Kaplan EL, Meier P (1958). Nonparametric estimation from incomplete observations. J Am Statist Assn.

[15] Montgomery AA, Graham A, Evans PH, Fahey T (2002). Inter-rater agreement in the scoring of abstracts submitted to a primary care research conference. BMC Health Serv Res.

[16] Li Y, Guo Y, Shen X, Liu H, Mei H, Xu H, Canavese F, Chinese Multi-center Pediatric Orthopedic Study group (2019). Radiographic outcome of children older than twenty-four months with developmental dysplasia of the hip treated by closed reduction and spica cast immobilization in human position: a review of fifty-one hips. Int Orthop.

[17] Hong K, Yuan Z, Li Y, Zhi X, Liu Y, Xu H, Canavvese F (2019). Femoral anteversion does not predict redislocation in children with hip dysplasia treated by closed reduction. Int Orthop.

[18] Li Y, Xu H, Slongo T, Zhou Q, Liu Y, Chen W, Li J, Canavese F (2018). Bernese-type triple pelvic osteotomy through a single incision in children over five years: a retrospective study of twenty eight cases. Int Orthop.

[19] Suh DH, Lee DH, Jeong WK, Park SW, Kang CH, Lee SH (2012). Virtual Bernese osteotomy using three-dimensional computed tomography in hip dysplasia. Arch Orthop Trauma Surg.

[20] Siebenrock KA, Schoeniger R, Ganz R (2003). Anterior femoro-acetabular impingement due to Acetabular retroversion. Treatment with periacetabular osteotomy. J Bone Joint Surg Am.

[21] Hayashi S, Hashimoto S, Matsumoto T, Takayama K, Shibanuma N, Ishida K, Nishida K, Kuroda R (2018). Computer-assisted surgery prevents complications during periacetabular osteotomy. Int Orthop.

[22] Matsunaga A, Akiho S, Kinoshita K, Naito M, Yamamoto T (2018). The prevalence and risk factors for delayed union of the superior pubic ramus at one year after curved periacetabular osteotomy: its risk factor and outcome. Int Orthop.

[23] Akiho S, Kinoshita K, Matsunaga A, Ishii S, Seo H, Nishio J, Yamamoto T (2018). Incidence of delayed union one year after peri-acetabular osteotomy based on computed tomography. Int Orthop.

[24] Tanaka T, Moro T, Takatori Y, Oshima H, Ito H, Sugita N, Mitsuishi M, Tanaka S (2018). Evaluation of the three-dimensional bony coverage before and after rotational acetabular osteotomy. Int Orthop.

[25] Hamada H, Takao M, Sakai T, Sugano N (2018). Morphological variation of the anterior inferior iliac spine affects hip range of motion in flexion after rotational acetabular osteotomy. Int Orthop.

